# Epizootic Diseases

**Published:** 1855

**Authors:** 


### EPIZOOTIC DISEASES.

The following are some extracts from a report made by the Governors of the Royal Veterinary College to the Council of the Royal Agricultural Society.

"Pleuropneumonia.——Although the cattle throughout the country suffered in this as in previous years from the epizootic affection, pleuropneumonia, still the losses have been by no means so great as on former occasions. The nature of this disease, when it becomes fully established in the system, is such that it will ever render ineffective the best curative measures which science can devise; but, notwithstanding this, there are many reasons for believing that preventive means may be had recourse to with decided benefit. From investigations made by their Professor during the past year, the Governors have no reason to alter the opinion expressed in a former report, that the inoculation of cattle will prove either a safe or an effectual preventive of pleuropneumonia. This system of inoculation is not based upon any known principle of medical science which regulates the *artificial* production of disease to give immunity to an animal against a *natural* attack of the same malady. The Governors are induced to make these remarks, from observing that attempts are again being made to introduce the system of inoculation, under the plea that it has proved highly advantageous in numerous instances on the Continent, and therefore it continues to be practised in France, Belgium, and Germany. With regard to the true principles of preventing the extension of pleuropneumonia, it cannot be doubted that these will be found in removing the diseased animals from the healthy, in altering the system of management and feeding which was in use when the malady first appeared, and in exhibiting to the animals which had been exposed to the contagion mild aperient, diuretic, and diaphoretic medicine, in succession, to rouse the emunctories of the body, and following up these remedies by tonic and stimulating agents, to invigorate the vital forces. This plan has been adopted in numerous instances with the best results, many animals being thereby saved which otherwise would have been sacrificed to the disease.

"Disease in Lambs.—On the subject of epizootics, the Governors have to remark that in the spring of last year a malignant form of aphtha appeared in several districts among the lambs, and destroyed very many of these animals. From this affection having been observed in a comparatively mild form, the year before, in a few isolated cases only, and from its great prevalence as well as malignancy in 1854, it is to be feared that it may take on the character of an epizootic, and thus add another to the list of these diseases which have been so destructive of late to our cattle and sheep. Numerous letters have been received from the members of the Society detailing the progress of this disease, and the great losses which they had sustained thereby. Professor Simonds has also personally investigated the causes which appear to favour the production of the malady; but much still remains to be done, as, in consequence of the disease having shewn itself under the most opposite circumstances of breed, age, vicissitudes of weather, locality, plan of management, &c., the evidence obtained is most conflicting. The Governors have, however, every reason to believe, that the mystery which envelopes the secondary causes of this affection will vanish before a rigid and scientific research. In the meantime, they have much pleasure in stating that the recommendations of their Professor with regard to the treatment of the animals, and which were necessarily varied in many cases from the differences in the predisposing causes, have been attended with a satisfactory result.

The French Government having requested the aid of the Agricultural Society, in investigating the nature of a disease which had appeared in Turkey among the cattle intended for the Crimea, Professor Simonds was at once placed in communication with M. Herbert, the Consul-General of France in London, with directions to furnish all information that could aid the French Government in their inquiry. The following allusion to this circumstance is made in the report:—

"Contagious Typhus.—As a preliminary step, and with the sanction of the Consul-General, Professor Simonds has prepared a list of questions to be forwarded to the several veterinary surgeons now with their regiments in the Crimea. These questions are annexed to this report; and the Governors trust that the Society will agree with them in the opinion that on answers being obtained, such an amount of information will be possessed on this subject as cannot fail, when acted upon, to be of essential service in effecting the desired object. It is likewise to be remembered, that the history of these epizootic affections shews that, when once they have taken root in any of the countries of Europe, they have spread with greater or lesser rapidity over the whole Continent, destroying the cattle by thousands in their course. It is this circumstance, added to the fact of the existence of the disease in Turkey, which has created so much solicitude on the part of the French Government, and the Governors cannot but feel equally anxious in the matter, seeing that if the cattle in France or Germany should become affected, our own would ere long fall victims to the direful pest, from the free importations of animals which now exists."

